# Annotations

**Published:** 1898-10-08

**Authors:** 


### ANNOTATIONS.

On the occasion of issuing our Special Autumn Number we take the opportunity of drawing attention to the fact that the publishers of The Hospital (The Scientific Press, Southampton Street, W.C.) are also publishers of medical and other scientific books, and would be glad at any time to confer with authors as to the production of their works.

At Kemptown, Brighton, a convalescent home for French patients has just been opened. It is in connection with the French Hospital in Shaftesbury Avenue, and will be conducted by the Sisters of the Sacred Heart. It is to receive a few old men and women as permanent residents, besides the patients who go there to complete their recovery. The building, which is of red brick, faces the sea, and is separated from the road by a good garden. The accommodation is for thirty persons. Ten old women and six old men will be admitted.

The first batch of the sick and wounded from the Soudan have arrived at Netley. The patients are principally those who have suffered on the line of march, and most are fever cases well on the road to convalescence. They came home on the s.s. "Rameses," which was comfortably fitted up for the sick. The first of the wounded actually from Omdurman were expected home on Thursday. Although no official announcement has yet been made, it is expected that the Queen will visit the sick and wounded soldiers at Netley.

The new Greig-Smith memorial operating theatre at the Bristol Royal Infirmary has just been opened by Sir William MacCormac. The late Mr. Greig-Smith devoted a large amount of time in preparing a schedule of alterations and innovations required to bring the facilities for operations up to date at the Bristol Royal Infirmary, and his proposals were adopted and in course of being carried out at the time of his death. It was then decided to still further complete the scheme by the addition of an ophthalmic theatre and institute, the whole as a memorial to Mr. Greig-Smith.

It is with regret that we announce the death of Mr. William Henry Hughes, for many years secretary of the Cancer Hospital, Brompton, at the early age of 45.

The foundation-stone of a cottage hospital for Castle Douglas has just been laid with Masonic honours by Sir Mark M'Taggart-Stewart. The hospital is to serve as a Diamond Jubilee memorial, and has been erected by public subscriptions.

It seems that there is much lack of accommodation for typhoid cases at Norwich just now. The beds set apart for typhoid fever at the Norfolk and Norwich Hospital are full. The isolation hospital does not admit such cases, and many patients who have been brought to the hospital have had to be sent back to their homes.

The Queen has nominated and appointed Sir William MacCormac, Bart., President of the Royal College of Surgeons, and Sir Francis Henry Laking, M.D., to be Knights Commander of the Royal Victorian Order; and Alfred Downing Fripp, Esq., and Fleet Surgeon Alfred Gideon Delmege, M.D., to be Members of the Fourth Class of the same Order, in recognition of their services in connection with the recent accident met with by His Royal Highness the Prince of Wales.

### An Anti Vivisection Meeting.

On the occasion of the Church Congress which was held in Bradford last week the Church Anti-Vivisection League organised a meeting which turned out to be a very tumultuous affair. The usual well-worn stories were trotted out, and some more than ordinarily curious reasons against operations upon animals were put forward. One speaker is reported to have said that "he held such a view of the scheme of creation that he regarded the animals as our brothers and sisters—(loud laughter)"; while another held that "the whole universe was a solidarity, and that to hurt any part of it must necessarily be to hurt themselves." The meeting, however, did not seem to find this very convincing, and a resolution which was proposed by Dr. Hime was carried by an overwhelming majority. The resolution, which seems to us to have been judiciously worded, was as follows: "That in the opinion of this meeting human interests are paramount in importance as compared with all other living creatures; and therefore we are justified in utilising the lower animals for the preservation of human health and life, all unnecessary suffering being avoided."

### The Control of Small-pox by Isolation.

Speaking last week at a conference of medical officers of health at Birmingham, Dr. John C. McVail pointed out that we have arrived at this point—that, as we can no longer rely on vaccination and revaccination being so universal as to afford protection from small-pox, we should have in future to trust to isolation; and he urged that, as against epidemic small-pox, isolation must turn out to be only a broken reed. In the condition in which we now live, with vaccination and revaccination partly prevalent, hospital isolation is a measure of very great value both with regard to the sporadic and the epidemic disease; but, if vaccination were entirely wanting, such hospitals would be almost useless, at any rate against the epidemic disease, which would almost certainly overleap all artificial bounds. Even if we were to grant the possibility of controlling small-pox by isolation, we must bear in mind what sort of an isolation it would be which would be necessary for such a purpose, to what sort of inconveniences the public would have to submit whenever small-pox broke out amongst them, and what an amount of hospital accommodation would have to be kept in constant readiness. It would not be a mere removal of the patient. "It means isolation of the poison by disinfection, or, still better, by the destruction of infected articles; it means not merely the removal of the patient, but his very early removal, and this involves search for persons who have been exposed to infection, and constant watching of such persons during the possible incubation of the disease." As regards interference with "liberty," isolation is a far bigger business than vaccination.

### The Sirdar's Despatch.

The despatch by the Sirdar, in which he describes the battle of Khartoum, contains the following reference to the Medical Department: "The Medical Department was administered with ability and skill by Surgeon-General Taylor, principal medical officer, who was well assisted by Colonel McNamara, whilst the medical organisation of the Egyptian army fully maintained its previous excellent reputation, under the direction of Lieut.-Colonel Gallwey and his staff. The general medical arrangements were all that could have been desired, and I believe the minimum of pain and maximum of comfort procurable on active service in this country was attained by the unremitting energy, untiring zeal, and devotion to their duty of the entire medical staff." The following officers and non-commissioned officers of the Royal Army Medical Corps are mentioned for good service: Lieut.-Colonel A. T. Sloggett (wounded), Lieut.-Colonel G. A. Hughes, Major C. A. Webb, Major G. Robinson. Major G. F. A. Smythe, Major D. Wardrop, Major R. W. Barnes, Major E. M. Wilson, Major A. Dodd, Major M. O'D. Braddell, Major C. R. Kilkelly, Major W. H. Pinches, Major H. M. Adamson, Major D. M. O'Callaghan, Major H. B. Mathias, Captain A. Y. Reily, Captain R. H. Penton, Captain H. E. Hill Smith, Captain C. S. Spong, Captain P. H. Whiston, Captain G. A. T. Bray, Captain J. W. Jennings, Captain H. N. Dunn, Lieutenant E. W. Bliss, Lieutenant S. L. Cummins, First-class Staff Sergeant Hoist, Sergeant Scrase.

### Window Accidents.

Attention has again been drawn to the frequency with which loss of life and serious injury occur from accidents in window cleaning. Probably it will never be possible to eliminate all danger from the work of cleaning the outsides of windows any more than from the painting and decoration of the outsides of houses, and with the growing fashion for lofty buildings, the probability is that, notwithstanding improved forms of window sashes, such accidents will increase. We would point out, then, that, so far as great towns are concerned, the windows are not alone to blame in this matter. There can be no doubt that in London they require cleaning at least three times as often as would be necessary were not the London atmosphere so highly charged with smoke, and that a very considerable proportion of the deaths from window cleaning must be put down to the account of the smoke fiend. Nor is it easy to see how the difficulty is to be got over for the griminess which so quickly renders our windows dark and useless does not result so much from the occasional outpouring of smoke from our electric light manufacturers and others—bad as that is from other points of view—but arises to a large extent from the daily and hourly fouling of the atmosphere by hundreds of thousands of domestic fires; and as the manufacture of domestic smoke is, we suppose, one of the prescriptive rights of the free-born Englishman, there seems but little chance of a serious diminution of the mischief—unless, indeed, the gas companies will kindly let us have gas at such a price that it shall be cheaper to use than coal, or some inventor will discover some method of burning coal without smoke, which shall be so simple, so cheap, and so effectual as to warrant our rulers in making its use compulsory. But the one solution seems almost as far off as the other.

### Plague, Cholera, and other Pestilences.

One must always look at these matters with a due sense of proportion. No one can deny that plague has caused a heavy loss of life in certain parts of India during the last couple of years. Still, if we compare the deaths from plague with those which have been going on all the time from other diseases, the presence of which has attracted but little attention and no excitement, we see how questionable is the policy, now, happily, being given up, of imposing severe restrictive measures, aimed at the suppression of one disease alone, instead of steadily enforcing those sanitary reforms which experience has shown to be most operative in lowering the general mortality. From its first appearance in India up to the present time, a period of about two years, plague has, it seems, carried off about 100,000 victims. But cholera, in 1896 alone, caused 471,779 deaths; small-pox, 141,443; and fevers, a term which, of course, includes the various forms of malaria, the enormous number of 4,578,944 persons. We have never felt surprised that the natives have protested against many of the coercive measures imposed in the name of the plague—measures which were, to all intents and purposes, that same quarantine against which England has again and again raised her voice in regard to other diseases; and we cannot feel altogether sorry that with the steady abandonment of restrictive plague measures, which seems to be going on in various parts of India, the mortality returns will in that country, as in others, be allowed to become again the indices of sanitary progress, instead of being twisted out of their course by purely temporary expedients. If communities choose to adopt proper sanitary precautions for the prevention of disease rather than for the mere segregation of cases after disease has broken out, they will in time attain, like their Western brethren, a comparatively low mortality; but if they choose to take a purely Eastern view of things, and if they prefer to keep their faiths, their customs, their castes, and their insanitary abominations they will get their reward. Truly, they will die a little sooner than they need do, but as to that each community must decide for itself. If they wish for Western mortality they must put aside many Eastern customs.

